# Prevalence and Correlates of Physical Comorbidities in Alcohol Use Disorder (AUD): a Pilot Study in Treatment-Seeking Population

**DOI:** 10.1007/s11469-021-00734-5

**Published:** 2022-01-23

**Authors:** P. V. AshaRani, Mohamed Zakir Karuvetil, Tan Yeow Wee Brian, Pratika Satghare, Kumarasan Roystonn, Wang Peizhi, Laxman Cetty, Noor Azizah Zainuldin, Mythily Subramaniam

**Affiliations:** 1 Research Division, Institute of Mental Health, 10 Buangkok View, Singapore, 539747 Singapore; 2 National Addictions Management Service, Institute of Mental Health, 10 Buangkok View, Singapore, 539747 Singapore; 3 Saw Swee Hock School of Public Health, National University of Singapore, Singapore, 117549 Singapore

**Keywords:** Alcohol use disorder, Liver diseases, Nicotine dependence, Chronic conditions, Physical comorbidities

## Abstract

This study aimed to understand the prevalence of physical comorbidities, undiagnosed and inadequately controlled chronic physical conditions and correlates of high cholesterol, hypertension and liver enzyme abnormalities in those with alcohol use disorder (AUD). Participants (*n* = 101) with AUD were recruited from a tertiary care centre through convenient sampling. The prevalence of physical and psychiatric comorbidities in the sample was 83.17% and 51.49%, respectively. Around 53.47% had two or more chronic physical conditions (multimorbidity). Hypertension (44.55%), asthma (23.76%), high cholesterol (22.77%) and liver enzyme abnormalities (21.78%) were the top four physical comorbidities. The prevalence of undiagnosed and inadequately controlled chronic physical conditions was 61.4% and 32.7%, respectively. Gender, education and body mass index (BMI) were associated with hyperlipidaemia while age and education were associated with hypertension. Higher waist-hip ratio was associated with liver enzyme abnormalities. Routine clinical care must include regular screening and follow-up of the risk groups to monitor their physical and mental health.

Alcohol use disorder (AUD) is one of the most prevalent psychiatric disorders globally with a 12-month prevalenceof 6.4% (Rehm & Shield, 2019). There are around 2.3 billion current drinkers in the world and over 60% of them indulge in heavy episodic drinking (World Health Organization, 2019). Alcohol accounts for 5.3% of total mortality and 5.1% of the global burden of diseases. Alcohol use is also associated with other substance use, psychiatric disorders, social dysfunction, homelessness, violence, suicide, homicide, loss of productivity and other physical comorbidities (Castillo-Carniglia et al., 2019; Compton et al., 2007; Curtis et al., 2019; Kiekens et al., 2021; Tanaree et al., 2017; World Health Organization, 2019).

Alcohol consumption is a strong risk factor for non-communicable diseases such as cardiovascular diseases, diabetes, cancer and liver diseases, which could affect the user’s life expectancy (Baliunas et al., 2009; Shield et al., 2020; World Health Organization, 2019). Those who consume 12–24 g of alcohol per day have a higher risk of developing liver diseases than non-drinkers (Rehm et al., 2010). The risk is 7 times higher in people who consume 50 g/day and > 26 times for those who take 100 g of alcohol per day (Bellentani et al., 1997). A systematic review and meta-analysis showed a twofold higher risk for cancer and heart diseases and 15 times higher risk for liver diseases in treatment-seeking AUD patients when compared to the general public (Roerecke & Rehm, 2013). Udo et al. (2015) showed that a lifetime history of AUD is associated with a higher risk for heart diseases and other chronic conditions even after stable remission, especially in the elderly. Mudd et al. (2020) in their study showed that individuals with AUD who have chronic conditions disengage from primary healthcare (decreased utilisation of medication and other services) and have higher utilisation of emergency services. This adversely affects the diagnosis of comorbidities and management of chronic conditions, thus leading to adverse treatment outcomes. It is clear that AUD is a leading cause of significant disability and morbidity, which is not because of the current use of the substances, but reflects the impact of lifetime use (Compton et al., 2007).

A nationwide study done in Singapore in 2016 found a lifetime prevalence of 4.1% and 0.5% for alcohol abuse and dependence, respectively (Subramaniam et al., 2020). Lifetime alcohol abuse showed an increase from 3.1 to 4.1% between 2010 and 2016 (Subramaniam et al., 2020). An earlier population-wide study conducted in 2010 in Singapore found that more than half of those with AUD (52.8%) had a chronic physical condition (Subramaniam et al., 2012). A high prevalence of chronic pain (23.6%), respiratory diseases (22.4%) and hypertension (17.4%) was observed in this population. Those with AUD also had higher odds of back problems, migraines, asthma, other respiratory conditions such as bronchitis emphysema, arthritis or rheumatism, gastric ulcers and back problems including problems with disc and spine than those without AUD (Subramaniam et al., 2012). In view of the rising number of AUD cases and data on the higher prevalence of chronic physical conditions at the population level, a clear evidence on the adverse health impact of AUD is essential.

While a lot of attention has been given to psychiatric comorbidities in AUD in clinical as well as population-level studies (Yang et al., 2018), not much is known about the prevalence and types of physical comorbidities in the treatment-seeking population. Piette et al. (1998) reported that compared to a community sample, those seeking treatment for AUD were more likely to suffer from chronic conditions during a 10-year follow-up period. However, there is a dearth of literature on this population, despite the adverse health and economic impact these comorbidities can have on the patients. It is unclear what proportion of the treatment-seeking population have physical comorbidities and if they are aware and seek treatment for their comorbidities. Therefore, a detailed understanding of the prevalence, the type of chronic physical conditions and factors influencing the comorbidities is warranted.

This pilot study aimed to understand the (a) prevalence of physical comorbidities in treatment-seeking populations with AUD, (b) the prevalence of undiagnosed and inadequately controlled cases of chronic physical conditions and (c) the factors associated with high cholesterol, hypertension and liver enzyme abnormalities, which are the common AUD-related conditions, in this population.

## Materials and Methods

### *Study Sample*

In this cross-sectional pilot study, 101 participants with AUD were recruited from the inpatient and outpatient clinics of a tertiary psychiatric care hospital in Singapore during October 2020 to January 2021. Adult participants with AUD were selected through convenient sampling. Participants were recruited through referrals by the attending clinicians or self-referrals, in response to the posters and brochures displayed at the clinic. Screening was done by the clinicians or trained study team members, and written informed consent was obtained from all eligible participants as per the approved ethics procedures. The survey was administered face to face by trained study team members. The eligibility criteria included age of ≥ 21 years (adults, as the legal age of majority is 21 years in Singapore); a diagnosis of AUD as per DSM-5 criteria; ability to speak either one of the four local languages: English, Chinese, Malay or Tamil; and sufficient mental capacity to do the study (as assessed by the attending clinicians). Participants were excluded if they had a comorbid substance use or gambling disorder.

### Sample Size

The sample size for this pilot study was estimated based on the ‘stepped sample size’ rule of thumb (Machin et al., 2018; Whitehead et al., 2016). Assuming the effect size to be extra small (< 0.1) and a power of 80% (significance at 5%), a sample size of 100 is recommended for pilot studies.

### Measures

#### Sociodemographic Information

The sociodemographic information captured included age, gender, ethnicity, education, marital status, employment status and income.

#### Anthropometric Measures

The height, weight, waist and hip circumference and blood pressure of the participants were measured on the day of their interview. The BMI was calculated as per the clinical practice guidelines where a cut-off value of 25 kg/m^2^, 30 kg/m^2^ and 18.5 kg/m^2^ qualified conditions for overweight, obesity and underweight, respectively (Lee et al., 2016). For regression analysis, two groups were used: normal BMI and overweight/obese. The group for underweight was excluded due to the small sample size (*n* = 6). The waist and hip circumference of an individual indicates the risk for diabetes and cardiovascular diseases (Parker et al., 2009). A waist-hip ratio of > 0.90 for men and > 0.85 for women indicates a risk of metabolic disorders (Health Promotion Board, 2016). A diagnosis of high blood pressure was given if the blood pressure exceeded 140–90 mm/Hg, as per the existing clinical guidelines (Ministry of Health, 2017).

#### Smoking and Nicotine Dependence

Smoking status was determined by a combination of two items on the self-reported questionnaire, (1) an item on their current smoking status (smoker, former smoker or nonsmoker) and lifetime cigarette consumption (at least 100 cigarettes in their lifetime to be considered a smoker or a former smoker) (Asharani et al., 2020). Those who scored ≤ 4 in the Fagerstrom Test for Nicotine Dependence (FTND) were classified as low dependence, while 5–7 was classified as moderate and ≥ 8 was classified as high dependence. Overall nicotine dependence was calculated if the score was ≥ 5.

#### Chronic Condition Checklist

The chronic condition checklist captured 18 different chronic conditions (Abdin et al., 2020) and an additional 3 conditions specific to AUD patients. The chronic conditions included asthma, diabetes, high blood pressure, arthritis, cancer, neurological conditions (epilepsy, convulsions), Parkinson’s disease, stroke or major paralysis, congestive heart failure, heart diseases, back problems including disc or spine, stomach ulcer, chronic inflamed bowel or enteritis or colitis, thyroid problems, kidney problems, chronic lung diseases, migraines and high cholesterol. The alcohol-specific chronic conditions included hepatitis B, hepatitis C and liver diseases (Novo-Veleiro et al., 2016). Participants were asked if a doctor had ever diagnosed them with any of these chronic conditions in their lifetime. Those who answered ‘yes’ were further probed for the age of diagnosis and if they had sought treatment for that specific condition in the past 12 months. The chronic conditions were also verified against the medical records and any missing details were captured.

#### Psychiatric Conditions

Psychiatric comorbidities of the participants were captured from the medical records and were grouped into schizophrenia spectrum and other psychotic disorders, bipolar and related disorder, depressive disorders, anxiety disorders, obsessive–compulsive and related disorders, trauma and stressor-related disorders, sleep–wake disorders and personality disorders.

#### Blood Tests

Blood tests were offered to the participants as a part of the study to assess the prevalence of undiagnosed and inadequately controlled chronic physical conditions. The tests offered included the lipid profile, liver function tests and glycosylated haemoglobin (HbA1c).

A value of ≥ 7% for HbA1c was taken as high (Ministry of Health, 2014). A diagnosis of hypercholesterolemia (high values of low-density lipoprotein (LDL) + total cholesterol (TC) was made when the value for TC and LDL was ≥ 5.2 mmol/L and ≥ 3.4 mmol/L, respectively. The reference range for hypertriglyceridemia used was ≥ 2.3 mmol/L (Ministry of Health, 2016). Dyslipidaemia was defined as high TC (≥ 5.2 mmol/L), LDL (≥ 3.4 mmol/L) and triglycerides (TG, ≥ 2.3 mmol/L), as per the Ministry of Health’s clinical guidelines (Ministry of Health, 2016). Liver enzyme abnormalities were diagnosed if gamma-glutamyl transferase (GGT) levels were above 40 U/L or alanine aminotransferase (ALT) or aspartate aminotransferase (AST) were > 36 U/L. For regression analysis, hyperlipidaemia (includes dyslipidaemia, hypercholesterolemia and hypertriglyceridemia) was used as the dependent variable.

Undiagnosed cases of chronic physical conditions included cases where no formal diagnosis was given previously for the conditions under study (as captured from the medical records or through self-report); however, the blood tests showed elevated levels that qualify a diagnosis. Inadequately controlled cases included those cases where the participant reported a pre-existing chronic physical condition and the results of the blood tests showed elevated levels that indicated inadequate management of the disease under study.

### Analysis

A descriptive analysis was performed on the sociodemographic variables and the prevalence of the chronic physical conditions was reported. Multivariate logistic regression was performed to study the factors associated with hypertension, hyperlipidaemia and liver enzyme abnormalities (dependent variables). Sociodemographic factors, waist-hip ratio, BMI and smoking status were used as independent variables. Asthma was excluded from the regression analysis as the majority of the cases were childhood asthma which occurred prior to their AUD. All the analyses were carried out using IBM SPSS Statistics 23.0 (IBM Corp, Armonk, NY, USA).

## Results

### Sociodemographic and Clinical Characteristics of the Sample

A total of 101 patients with AUD were enrolled in the study. The majority of the participants were male 84.16% (*n* = 85), of Indian ethnicity (59.41%, *n* = 60), aged 50 or above (52.48%, *n* = 53), single/unmarried/divorced (57.43%, *n* = 58) and had a monthly income below S$2,000 (66.34%, *n* = 67). Around 38.61% (*n* = 39) had normal BMI, 36.63% (*n* = 37) were overweight and 17.82% (*n* = 18) were obese. Around 78.22% (*n* = 79) had waist-hip ratio above the normal range. The majority of the sample (67.33%, *n* = 68) were current smokers. The prevalence of ND for current smokers and overall sample were 42.64% (scores 5–10) and 28.71%, respectively. The detailed information is indicated in Table 1.

**Table 1:** Sociodemographic and clinical characteristics of the patients

Variables	*n*	%
Age group		
21–49	48	47.52
^ a^50 and above	53	52.48
Gender		
Male	85	84.16
Female	16	15.84
Ethnicity		
Chinese	28	27.72
Malay	4	3.96
Indian	60	59.41
Others	9	8.91
Education		
Primary and below	37	35.92
Secondary	33	32.03
Tertiary	31	30.10
Marital status		
Married/cohabiting	43	42.57
Single/unmarried/widowed	58	57.43
Employment		
Employed	51	50.50
Others	50	49.50
Monthly income		
Below 2000	67	66.34
2000 and above	34	33.66
Monthly household income		
Below 10,000	79	78.22
10,000 and above	22	21.78
BMI		
Normal range	39	38.61
Underweight	6	5.94
Overweight	37	36.63
Obese	18	17.82
Missing	1	1.00
Waist-hip ratio		
Normal	22	21.78
Exceed	79	78.22
Psychiatric comorbidities		
Yes	49	48.51
No	52	51.48
Number of chronic conditions		
None/no comorbid condition	17	16.83
One chronic physical condition	30	29.70
Two or more chronic physical conditions	54	53.47
Smoking		
Current smokers	68	67.33
Non-smokers/past smokers	33	32.67
Nicotine dependence		
Low	39	38.61
Moderate	19	18.81
High	10	9.90
^b^Blood pressure		
Normal	95	94.06
High blood pressure	6	5.94

^a^Upper age limit of the participant, 74 years; ^b^measured on the day of recruitment

### Prevalence of Psychiatric Comorbidities

Around 48.51% (*n* = 49) had no psychiatric comorbidities, 39.60% (*n* = 40) had one, 8.91% (*n* = 9) had two and 2.97% had 3 or more psychiatric comorbidities. Depressive disorders (31.68%, *n* = 32) followed by trauma and stress-related disorders (12.87%, *n* = 13), schizophrenia spectrum and other psychotic disorders (6.93%, *n* = 7), anxiety disorders (6.93%, *n* = 7), personality disorders (4.95%, *n* = 5), bipolar and related disorders (1.98%, *n* = 2) and sleep–wake disorders (0.99%, *n* = 1) were the most prevalent psychiatric conditions. There were no cases of obsessive–compulsive and related disorders in the study sample.

### Prevalence of Physical Comorbidities

The prevalence of physical comorbidity in the sample was 83.17%. The majority of the sample had 2 or more physical comorbidities (multimorbidity, 53.47%, *n* = 54), 29.70% (*n* = 30) had one and 16.83% (*n* = 17) had no physical comorbidities. The most prevalent chronic physical condition was hypertension (44.55%, *n* = 45), followed by asthma (23.76%, *n* = 24), hyperlipidaemia (22.77%, *n* = 23) and liver diseases (21.78%, *n* = 18). The detailed results regarding the chronic physical conditions are indicated in Table 2. The median age of diagnosis of asthma was 12 years (mean 12.8 years, *SD* 11.82) and only half of the sample sought treatment for the condition in the past 12 months. The median age of diagnosis of hypertension was 50 years (mean 47.25 years, *SD* 9.88) and 75.6% of these subjects sought treatment in the past 1 year. Likewise, the ages of diagnosis of hyperlipidaemia and liver enzyme abnormalities were 54 years (mean 51.15 years, *SD* 9.98) and 46 years (mean 45.79 years, *SD* 11.91), respectively. 69.57% of those with hyperlipidaemia and 40.91% of those with liver enzyme abnormalities had sought treatment for their condition. The prevalence of undiagnosed conditions was 61.38% (*n* = 62), of which the highest prevalence was observed for liver enzyme abnormalities (45.54%, *n* = 46), followed by high cholesterol (14.85%, *n* = 15). The prevalence of inadequately controlled chronic physical conditions was 32.67% (*n* = 33). Liver enzyme abnormalities (12.87%, *n* = 13) were the most prevalent condition in this category (Table 2).

**Table 2:** Prevalence of chronic physical conditions in the sample

Chronic physical conditions	*n*	%	Age of diagnosis (median)	Sought treatment in the past 12 months (%)
Overall prevalence				
Hypertension or high blood pressure	45	44.55	50	75.56
Asthma	24	23.76	12	50.00
Hyperlipidaemia or high cholesterol	23	22.77	54	69.57
Any liver disease	22	21.78	46	40.91
Back problems including disc or spine	18	17.82	35	33.33
Diabetes	16	15.84	45	93.75
Arthritis or rheumatism	12	11.88	43	33.33
Stomach ulcer	12	11.88	42	50
Migraine headaches	12	11.88	23	50
A neurological condition, such as epilepsy or convulsions	11	10.89	10	54.55
Hepatitis C	11	10.89	45	45.45
Stroke or major paralysis (inability to use arms or legs)	9	8.91	54	66.67
Heart disease (including a heart attack, coronary heart disease, angina or other heart diseases)	9	8.91	49	66.67
Chronic inflamed bowel, enteritis or colitis	4	3.96	49	50
Chronic lung diseases such as chronic bronchitis or emphysema (excluding asthma)	4	3.96	55	50
Thyroid disease	3	2.97	62	33.33
Hepatitis B	3	2.97	51	0
Parkinson’s disease	2	1.98	62	100
Kidney failure	2	1.98	55	0
Cancer diagnosis	1	0.99	-	-
Congestive heart failure	0	0	-	-
Undiagnosed chronic physical conditions				
Prevalence of undiagnosed chronic physical conditions	62	61.38	-	-
High cholesterol	15	14.85	-	-
* 1. Dyslipidaemia*	3	2.97	-	-
* 2. Hypercholesterolemia*	11	10.89	-	-
* 3. Hyper trigliceridemia*	1	0.99	-	-
Liver enzyme abnormalities	46	45.54	-	-
Diabetes	0	0	-	-
Hypertension	1	0.99	-	-
Inadequately controlled chronic physical conditions				
Prevalence of inadequately controlled chronic physical conditions	33	32.67	-	-
High cholesterol	4	3.96	-	-
* 1. Dyslipidaemia*	0	0.0	-	-
* 2. Hypercholesterolemia*	1	0.99	-	-
* 3. Hyper trigliceridemia*	3	2.97	-	-
Liver damage	13	12.87	-	-
Diabetes	7	6.93	-	-
Hypertension	5	4.95	-	-

### Factors Associated with Hyperlipidaemia, Hypertension and Liver Enzyme Abnormalities

The results of the logistic regression are presented in Table 3. Gender, education and BMI were significantly associated with hyperlipidaemia. Females (*OR* 0.07, 95% *CI* 0.01–0.9, *p* = 0.039) and those who had a higher education status (secondary school compared to primary school or below education, *OR* 0.03, 95% *CI* 0.003–0.3, *p* = 0.003) had lower odds of hyperlipidaemia. Those who were overweight/obese had higher odds (*OR* 24.09, 95% *CI* 2.60–223.26, *p* = 0.005) of developing hyperlipidaemia as compared to those with normal BMI. Age and education were significantly associated with hypertension. Those who were > 50 years (*OR* 3.39, 95% *CI* 1–11.30, *p* = 0.047) had higher odds of hypertension compared to those below 50 years. Compared to those who had an education status of primary school or below, those with secondary education had lower odds of hypertension (*OR* 0.26, 95% *CI* 0.07–0.96, *p* = 0.043). Compared to those with a normal waist-hip ratio, those with a higher ratio had higher odds of liver enzyme abnormalities (*OR* 3.44, 95% *CI* 1.07–11.07, *p* = 0.038).

**Table 3:** Factors associated with hyperlipidaemia, hypertension and liver enzyme abnormalities

	Hyperlipidaemia				Hypertension				Liver enzyme abnormalities			
	Odds ratio	*p* value	95% *CI*		Odds ratio	*p* value	95% *CI*		Odds ratio	*p* value	95% *CI*	
			*Lower*	*Upper*			*Lower*	*Upper*			*Lower*	*Upper*
**Age group**												
21–49 *(Ref)*												
50 and above	4.34	.10	0.74	25.53	**3.39**	**.047**	**1.02**	**11.30**	1.90	.32	0.53	6.76
**Gender**												
Male *(Ref)*												
Female	**0.07**	**.039**	**0.01**	**0.87**	0.19	.060	0.03	1.07	1.37	.69	0.30	6.15
**Ethnicity**												
Chinese *(Ref)*												
Malay	0.63	.80	0.02	24.56	5.39	.227	0.35	83.08	0.28	.33	0.02	3.58
Indian	0.91	.92	0.13	6.29	1.29	.685	0.38	4.32	1.76	.34	0.55	5.63
Others	2.18	.59	0.13	37.86	1.97	.480	0.30	12.98	0.55	.51	0.09	3.33
**Education**												
Primary and below *(Ref)*												
Secondary	**0.03**	**.003**	**0.03**	**0.30**	**0.26**	**.043**	**0.07**	**0.96**	0.94	.92	0.25	3.49
Tertiary	0.09	.11	0.01	1.75	0.57	.521	0.10	3.18	1.25	.80	0.22	7.05
**Marital status**												
Married/cohabiting *(Ref)*												
Others	1.02	.98	0.22	4.76	0.62	.377	0.21	1.80	1.35	.58	0.47	3.91
**Employment**												
Employed *(Ref)*												
Others	1.76	.53	0.30	10.16	2.53	.172	0.67	9.59	0.47	.23	0.13	1.63
**Monthly income**												
Below 2000 *(Ref)*												
2000 and above	0.14	.13	0.01	1.76	1.13	.872	0.25	5.18	0.69	.62	0.16	2.97
**Monthly household income**												
Below 10,000 *(Ref)*												
10,000 and above	2.36	.46	0.25	22.40	3.06	.104	0.79	11.82	0.80	.75	0.21	3.08
**BMI**												
Normal range *(Ref)*												
Overweight/obese	**24.09**	**.005**	**2.60**	**223.26**	2.09	.187	0.70	6.27	0.92	.88	0.32	2.67
**Psychiatric comorbidity**												
No												
Yes	2.54	.24	0.54	12.03	1.37	.536	0.51	3.72	0.69	.47	0.26	1.87
**Waist-hip ratio**												
Normal												
Exceed	1.21	.85	0.18	8.12	0.82	.756	0.22	2.96	**3.44**	**.038**	**1.07**	**11.07**
**Smoking**												
Current smokers *(Ref)*												
Non-smokers/past smokers	5.27	.41	1.07	25.98	1.61	.410	0.52	4.96	0.37	.08	0.13	1.11

Entries in bold indicate statistically significant results

## Discussion

This pilot study explored the lifetime prevalence of chronic physical conditions in patients with AUD and identified a prevalence of 83.17%, with high blood pressure (44.55%), asthma (23.76%), hyperlipidaemia (22.77%) and liver enzyme abnormalities (21.78%) being the top four diseases. This prevalence observed in the study is higher than that of the general population in Singapore, where the prevalence of hypertension, hyperlipidaemia and asthma was 13.2%, 15.3% and 10.6%, respectively (Subramaniam et al., 2020).

Alcohol use is associated with high blood pressure (Taylor et al., 2009), especially systolic blood pressure (Arkwright et al., 1982; Klatsky & Gunderson, 2008). Studies have shown a reversible relationship between AUD and hypertension where those with heavy drinking had 4 times higher blood pressure than non-drinkers and past drinkers, which was reversed upon abstinence (Arkwright et al., 1982; Gordon, 1983). It is unclear how alcohol affects blood pressure. It is believed to be due to its widespread biological impact on both the circulatory and nervous systems (Puddey et al., 2019). Alcohol use can not only lead to hypertension, but also worsen the management of the condition by interfering with the pharmacokinetics of the medications in diagnosed individuals, predisposing them to further negative health outcomes (Miller et al., 2005).

We also observed hyperlipidaemia (22.77%), a high prevalence of overweight/obesity (54.45%) and a higher wait-hip ratio (78.22%) in our patients. Several studies have reported an association between alcohol consumption, hyperlipidaemia, overweight/obesity and BMI (Shen et al., 2014; Steiner & Lang, 2017; Wakabayashi, 2013). Although the exact pathomechanism linking AUD and hyperlipidaemia is unclear, animal studies have shed light on the possible pathogenesis of these complications. Wang et al. (2010) showed that chronic alcohol consumption in rats was associated with liver injury which led to upregulation of specific genes in the cholesterol biosynthesis pathway leading to hyperlipidaemia. Dysregulated lipid metabolism in AUD leads to adipose tissue lipolysis and excessive storage of fat in skeletal muscles and other organs which is observable as high waist-hip ratio and higher BMI in these individuals (Steiner & Lang, 2017). A longitudinal study among those who consume alcohol showed that heavy (≥ 30 gm/day) or stable drinkers gained weight over the 5-year follow-up period and had the highest prevalence of BMI (Wannamethee and Shaper, 2003). BMI is linked to liver diseases in those with AUD as excessive accumulation of fats in the liver will eventually lead to liver diseases (Steiner & Lang, 2017). Population-wide studies have further evidenced that high cholesterol and obesity together with alcohol use significantly increase the likelihood of severe liver diseases (Åberg et al., 2018; Pose et al., 2021) and premature death (Larsson et al., 2020; Younossi et al., 2019; Yusuf et al., 2020). A larger proportion of the participants (59.41%) were Indians who are at a higher risk of obesity (Hughes et al., 1997) and alcohol use (Abdin et al., 2014).

The prevalence of undiagnosed chronic physical conditions was 61.38% in the sample which mainly included liver enzyme abnormalities (45.54%) and high cholesterol (14.85%). No studies have been conducted till date in those with AUD for undiagnosed conditions. Hence, a comparison is not possible. However, the higher incidence of undiagnosed cases of liver enzyme abnormalities (45.54%) is of concern. This could be a result of heavy and chronic drinking in the patients and being unaware of their condition might lead to further deterioration due to continuous drinking. Chronic liver injury and inflammation can eventually lead to alcoholic fatty liver, steatohepatitis, alcoholic hepatitis, cirrhosis and cancer (Seitz et al., 2018). In Singapore, 0.9% of years of life lost (YLL) was due to cirrhosis of the liver (Singapore Burden of Disease & Injury Study Working Group, 2014). This is the second most common cause of mortality and morbidity annually in those with AUD, the primary reason being injuries (World Health Organization, 2019). In the current practice, patients are not screened regularly for AUD at the primary care settings and therefore there is a substantial treatment gap between the onset of alcohol problems and the point of intervention at the specialised treatment centres (Altamirano et al., 2012) which could worsen their liver conditions as well (Scafato et al., 2020). Therefore, regular screening should ideally start at the primary care settings for diagnosing AUD and associated comorbidities to avoid adverse health outcomes.

The current study showed that around 67.33% of the sample were current smokers and 28.71% had moderate to high dependence to nicotine. This is higher than the prevalence and nicotine dependence that is reported in the general population (16.1% and 3.3%) (Shahwan et al., 2019), and in those with mental illness (39.5% and 23.2%), in Singapore (Asharani et al., 2020). Smoking is one of the most common causes of mortality and morbidity worldwide (World Health Organization, 2019) and could add additional treatment burden in those with AUD by predisposing them to smoking-related diseases (Beynon et al., 2016). A study that compared the impact of smoking, AUD alone and as co-occurring disorders showed higher rates of homelessness, liver diseases, other substance use and disabilities in those with co-occurring disorders (MacLean et al., 2018). Those with smoking and co-occurring alcohol use have a higher risk of hyperlipidaemia and cardiovascular complication than with single disorder (Li et al., 2018; Vignesh et al., 2020). Smoking cessation should be promoted in this population to improve their treatment outcomes and quality of life.

Alcohol use is a cause for many of the mental and behavioural health conditions (Shield et al., 2013). AUD co-occurs with serious mental illness (SMI) and more than 20% of those with SMI suffer from AUD (Westreich, 2005). This study showed that 51.49% of the sample had at least one psychiatric comorbidity which is in agreement with previous reports (Tanaree et al., 2017). Drake et al. (Drake et al., 1991) in their literature review on the dual diagnosis of AUD and SMI reported that those with SMI use alcohol to cope with the symptoms of SMI and alleviate the side effects of psychiatric medication and for the avoidance of the ‘mentally ill’ labelling. These are the same reasons for smoking among those with SMI. We have noted a higher prevalence of depressive disorder (31.7%) in the study sample which corroborates other studies (Shield et al., 2013; Westreich, 2005). Depressive disorders are causally linked to AUD and the risk increases by 2–3 times in subjects with alcohol dependence (Rehm et al., 2003). Dual diagnosis also leads to worsening or relapse of SMI and adverse impacts on family, finance, health and legal and can lead to other substance use (Drake et al., 1991). Those with a dual diagnosis require more specialised care to treat both conditions concurrently (Westreich, 2005). While abstinence improves the treatment efficiency for depressive disorder, it does not guarantee recovery from the condition. Therefore, an integrated mental health and AUD treatment that engages the patient long term to prevent relapses or promote low-risk drinking of alcohol use would be beneficial.

We have also noted that gender (females compared to males), lower education and higher BMI were associated with hyperlipidaemia while the latter two were associated with hypertension. Wakabayashi and Araki (2010) studied the effect of age and gender on atherosclerosis risk where they evaluated the risk of developing hyperlipidaemia and hypertension in those who consume alcohol. The authors concluded that age and gender influence the risk of atherosclerosis through their influence on blood pressure and lipids in those with moderate to heavy alcohol use. A Mendelian randomisation study has shown a likely causal relationship with education and alcohol consumption where those with lower education had higher consumption of alcohol per occasion, binge drinking and frequency of alcohol-related negative consequences (Rosoff et al., 2021). Our findings corroborate this result where those with higher education (secondary school) had a lower likelihood of hyperlipidaemia and hypertension compared to those with an education status of primary school and below. Jung and colleagues (2002) further confirmed this correlation in a population-level study where the authors recorded that lower education was associated with hypertension. Age was associated with higher odds of hypertension. The National epidemiological survey on alcohol and related conditions (Grant et al., 2017) showed a higher drinking frequency, excessive drinking and AUD in older adults and those with lower education. Heavy drinking is associated with hypertension (Taylor et al., 2009), liver problems (Rehm & Roerecke, 2015), stroke (Patra et al., 2010) and cardiovascular diseases (Rehm et al., 2016). Given that alcohol use is associated with higher rates of mortality due to hypertension, stroke and cardiomyopathy, for which cholesterol is a modifiable risk factor, regular screening and management of these physical comorbidities in patients with AUD is imperative.

This study has several limitations. We have recruited the participants through a convenience sampling which is subjected to selection bias, thus affecting the generalisability of the results. Additionally, the chronic condition checklist used in the study collected data based on self-report. Thus, it may be subject to recall bias. While medical records were also used to corroborate the participants’ self-report, the medical records used were limited to institutional records. The National Electronic Health Records (NEHR) which is an integrated system of medical records across various hospitals across the country was not accessible for research purposes due to patient confidentiality concerns. The attending clinician at the study site might not have always transferred the information on chronic conditions from the NEHR into the institutional medical records. Thus, there is a possibility of missing information related to their chronic physical conditions. Additionally, the institutional medical records do not always capture the information on age of diagnosis or if treatment was sought in the past 12 months which contributed to a small subset of participants with missing data on these two variables. We did not capture the details of concomitant psychiatric medication use and the severity of AUD which can be confounding factors.

There are several clinical implications of these findings. The higher prevalence of diagnosed and undiagnosed physical comorbidities highlights a previously overlooked need in the routine clinical care of those with AUD. Hyperlipidaemia and hypertension are the common risk factors for cardiovascular diseases and death, and a higher proportion of these conditions in this group indicates an impending risk of mortality. Liver enzyme abnormalities, although are known complications of alcohol use, are not diligently monitored in all the patients. Lastly, the high prevalence of smoking adds an additional treatment burden to the management of these patients. In the past few years, treatment for AUD has focused on a harm reduction approach rather than complete abstinence; however, the recent evidence suggests that there is no safe limit recommendation for alcohol (Griswold et al., 2018). Therefore, complete abstinence must be the focus of the treatment, especially for those with a higher risk of alcohol-induced harms (Griswold et al., 2018). The specific sociodemographic groups who are at a higher risk of these comorbidities must be identified and followed up to avoid adverse health outcomes. This is of paramount importance in the coming years as the world is seeing a surge in AUD and liver diseases during the COVID-19 pandemic (Ahmed et al., 2020). This is believed to be due to the safe distancing measures and lockdown/stay home notices that triggered relapse to AUD in many people due to the disruption of their non-alcohol-related leisure activities, sports, boredom and social influences through social media platforms (Da, et al., 2020). In addition, the pandemic also had a negative impact on the mental health of the population that might have triggered increased alcohol consumption and relapse (Da et al., 2020). The COVID-19 infection is detrimental to those with AUD as they suffer from multiple comorbidities, low immunity and lack of social support (Da et al., 2020). Therefore, care providers should establish a referral network to identify those at risk and implement strategies to reduce the harm and to provide seamless care during this period.

## Conclusion

This pilot study showed a high prevalence of physical and psychiatric comorbidities in those seeking treatment for AUD. Hypertension, asthma, high cholesterol and liver enzyme abnormalities were the common physical comorbidities. A high prevalence of undiagnosed conditions was noted with liver enzyme abnormalities being the most common condition. The prevalence of smoking was higher in this group compared to the general population and those with mental illness, adding an additional treatment burden for this group. Female gender, higher education and a BMI within normal range were associated with a lower likelihood of hyperlipidaemia, whereas younger age and higher education had a lower likelihood of hypertension. A higher waist-hip ratio was associated with liver enzyme abnormalities. The results highlight the importance of routine screening in the clinical setting to detect any undiagnosed or inadequately controlled physical comorbidities early; to deliver timely treatment, integrated care; and to improve the wellbeing of the patient.
